# Temozolomide protects against the progression of glioblastoma via SOX4 downregulation by inhibiting the LINC00470‐mediated transcription factor EGR2

**DOI:** 10.1111/cns.14181

**Published:** 2023-03-29

**Authors:** Wenyang Li, Ming Wang, Wenjia Ma, Ping Liu, Mingming Zhang, Jiarong He, Yan Cui

**Affiliations:** ^1^ Department of Neurosurgery The Second Xiangya Hospital of Central South University Changsha China; ^2^ Department of Oncology The Second Xiangya Hospital of Central South University Changsha China

**Keywords:** angiogenesis, apoptosis, autophagy, early growth response 2, glioblastoma, LINC00470, SRY‐related high‐mobility‐group box 4, temozolomide

## Abstract

**Objective:**

Temozolomide is extensively applied in chemotherapy for glioblastoma with unclear exact action mechanisms. This article seeks to address the potential molecular mechanisms in temozolomide therapy for glioblastoma involving LINC00470.

**Methods:**

Bioinformatics analysis was conducted to predict the potential mechanism of LINC00470 in glioblastoma, which was validated by dual‐luciferase reporter, RIP, ChIP, and RNA pull‐down assays. LINC00470 expression and the predicted downstream transcription factor early growth response 2 (EGR2) were detected in the collected brain tissues from glioblastoma patients. Following temozolomide treatment and/or gain‐ and loss‐of‐function approaches in glioblastoma cells, cell viability, invasion, migration, cycle distribution, angiogenesis, autophagy, and apoptosis were measured. In addition, the expression of mesenchymal surface marker proteins was assessed by western blot. Tumor xenograft in nude mice was conducted for in vivo validation.

**Results:**

Mechanistic analysis and bioinformatics analysis revealed that LINC00470 transcriptionally activated SRY‐related high‐mobility‐group box 4 (SOX4) through the transcription factor EGR2. LINC00470 and EGR2 were highly expressed in brain tissues of glioblastoma patients. LINC00470 and EGR2 mRNA expression gradually decreased with increasing concentrations of temozolomide in glioblastoma cells, and SOX4 expression was reduced in cells by temozolomide and LINC00470 knockdown. Temozolomide treatment induced cell cycle arrest, diminished cell viability, migration, invasion, and angiogenesis, and increased apoptosis and autophagy in glioblastoma, which was counteracted by overexpressing LINC00470 or SOX4 but was further promoted by LINC00470 knockdown. Temozolomide restrained glioblastoma growth and angiogenesis in vivo, while LINC00470 or SOX4 overexpression nullified but LINC00470 knockdown further facilitated these trends.

**Conclusion:**

Conclusively, temozolomide repressed glioblastoma progression by repressing the LINC00470/EGR2/SOX4 axis.

## Graphical abstract

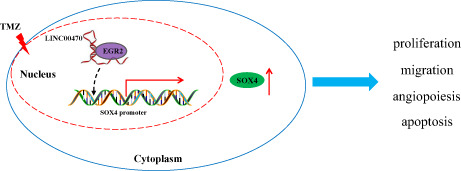

## 1 INTRODUCTION

Glioblastoma, which is believed to be generated by progenitor or neuroglial stem cells, has the highest degree of malignancy among gliomas and the highest prevalence among brain tumors.^1^, ^2^ Glioblastoma exhibits cell and nuclear rudimentary, microvascular proliferation, a high degree of cellularity, necrosis, and mitosis under the microscope.^3^ Glioblastoma appears in any age group with increasing incidence with age (median age of diagnosis is around 65 years old) and is more frequent in men.^4^ As of 2005, the Stupp protocol, consisting of surgery excision combined with adjuvant temozolomide chemotherapy and radiotherapy, has served as the care standard for glioblastoma.^5^ In contrast to radiotherapy treatment alone, incorporation with temozolomide resulted in improvement in overall and progression‐free survival of patients with glioblastoma.^6^ Temozolomide is an orally administered front‐line chemotherapy drug for patients with glioblastoma, whose action mode is primarily based on alkylating guanine residues N‐7 or O‐6 or adenine residues N‐3 within DNA, thus resulting in mismatching in the subsequent replication of DNA and in turn leading to cell cycle arrest, senescence, apoptosis, autophagy, and death.^7^, ^8^ Although the action mechanism of temozolomide in glioblastoma has been extensively researched, the concrete molecular mechanism of its impact in glioblastoma warrants in‐depth exploration.

It is well known that various dysregulated long noncoding RNAs (lncRNAs) are linked to glioblastoma progression with its involvement in nearly all tumor characteristics, comprising cell proliferation, invasion, angiogenesis, migration, severity, stemness, recurrence, and chemoresistance.^9^ LINC00470, a novel lncRNA localized at chromosome 18p11.32, is already proposed to serve as an oncogene in melanoma and endometrial cancer.^10^, ^11^, ^12^ LINC00470 was described as an oncogenic lncRNA in glioblastoma and a novel AKT activator that induces malignant features of glioblastoma, including cell proliferation and autophagy.^13^ Bioinformatics analysis in our research predicted that LINC00470 orchestrated a transcription factor, early growth response 2 (EGR2), and that SRY‐related high‐mobility‐group box 4 (SOX4) was a downstream target gene of EGR2. EGR2 is a transcription factor that participates in various cell processes, such as cell proliferation and cell cycle.^14^ EGR2 was demonstrated to serve as a carcinogenic gene in renal cell cancer by modulating cancer cell proliferation and migration.^15^ A prior publication reported that EGR2 knockdown curtailed cell invasion in glioma.^16^ EGR2 may promote glioblastoma in view of these findings. SOX4 is a carcinogenic gene that enhances stemness, cancer cell migration, metastasis, epithelial–mesenchymal transition, survival, and angiogenesis.^17^ Ikushima et al. observed that SOX4 was overexpressed in glioma‐initiating cells and was a key element in maintaining the stemness of glioma‐initiating cells.^18^ Therefore, we hypothesized that temozolomide may delay the development of glioblastoma by inhibiting the transcriptional activation of SOX4 through LINC00470‐regulated EGR2. This study is dedicated to delve into the impact of the relationship between temozolomide and the LINC00470/EGR2/SOX4 axis on glioblastoma progression, thus presenting a fresh theoretical basis for the treatment of glioblastoma with temozolomide.

## 2 MATERIALS AND METHODS

### 2.1 Ethical statements

This study that involved humans was ratified by the Medical Ethics Committee of The Second Xiangya Hospital of Central South University, and all patients signed an informed consent form. All animal experiments complied with the regulations and codes of practice for laboratory animal management and ethical requirements related to laboratory animals.

### 2.2 Clinical specimens

Brain tissues were harvested from 30 patients with glioblastoma at The Second Xiangya Hospital of Central South University between November 2016 and November 2017 and normal brain tissue sections (10 cases) (traumatic brain tissue) collected as controls. All samples were rapidly frozen in liquid nitrogen until further use. Inclusion criteria were as follows: patients with complete clinical information (including general condition, pathological diagnosis, treatment plan, survival, and follow‐up information) and without combined history of other malignancies. Exclusion criteria were as follows: patients with incomplete clinical data (no pathological diagnosis and immunohistochemistry [IHC] result) or the combined history of other malignancies.

### 2.3 Immunohistochemistry

The prepared sections were placed in boiling water with ethylene diamine tetraacetic acid (EDTA) buffer (pH = 9.0) for 10 min antigen retrieval, naturally cooled, and then washed 3 times with phosphate‐buffered saline (PBS). The sections were incubated in 3% hydrogen peroxide solution for 10 min to block endogenous peroxidase, washed 3 times in PBS, and blocked in serum for 30 min. Then, the sections were probed overnight at 4°C with 50 μL sealing solution‐prepared primary antibodies (Abcam) against Ki67 (ab15580, 1:100), vascular endothelial growth factor (VEGF, ab32152, 1:100), light chain 3 (LC3, ab232940, 1:100), and p62 (ab109012, 1:100), followed by three PBS washes. After being probed with secondary antibody for 0.5 h at room temperature, the sections were washed 3 times with PBS, developed with diaminobenzidine, and stained for 3 min with hematoxylin. Subsequently, the sections underwent 1–3 s of color separation with 1% ethanol hydrochloride and running water washing. Afterward, the sections were returned to blue with 0.6% ammonia and washed with running water. Finally, the sections were dehydrated with gradient ethanol, transparentized with xylene, sealed with neutral resin, and observed and photographed by an ordinary optical microscope.

### 2.4 Cell culture and treatment

Temozolomide were attained from Sigma‐Aldrich (T2577). LN229 and U87 cells (iCell Bioscience) were cultured with high‐glucose Dulbecco's modified Eagle's medium (DMEM; Gibco) encompassing 10% fetal bovine serum, and GL261 + luc cells (the mouse glioma cells with luciferase gene; iCell Bioscience) were cultured in the GL261 + luc cell‐specific medium (iCell‐0059a‐001b, iCell Bioscience). All of the above media contained 100 U/mL penicillin and 100 μg/mL streptomycin, and all of the cells were cultured in a constant temperature incubator (37°C, 5% CO_2_, and 95% humidity). Cells in the logarithmic phase were harvested for the experiments.

LN229, U87, and GL261 + luc cells were transfected with small interfering RNA (si)‐LINC00470 and lentiviral vectors overexpressing LINC00470, EGR2, and SOX4 (LV‐LINC00470, LV‐EGR2, and LV‐SOX4) (2.5 μg for each; all from GenePharma) strictly as per the manuals of a Lipofectamine 2000 transfection kit (Thermo Fisher Scientific). The sequences of siRNAs are detailed in Table S1. After transfection, cells were added to serum‐free DMEM for 48‐h further culture in a 5% CO_2_ and 37°C constant temperature incubator.

### 2.5 Cell counting kit‐8 assay

Glioma cells were seeded onto 96‐well plates at a density of 4000–6000 cells per well, followed by supplementation of fresh medium with the appropriate concentration of reagents in the next day. Following 48‐h incubation, cell viability was evaluated using cell counting kit (CCK)‐8 kits (KeyGEN Biotech) as per the protocols.

### 2.6 Quantitative reverse transcription polymerase chain reaction

Total RNA was extracted following the instructions of the Trizol kits (Invitrogen) and reversely transcribed into cDNA using the PrimeScript RT kit (RR037A; Takara). The samples were subjected to fluorescent PCR on a real‐time quantitative fluorescent PCR instrument system (ABI7500; Applied Biosystems) in accordance with the manuals of the TB Green® Premix Ex Taq™ II kit (RR820A, TaKaRa). The relative expression of each target gene was calculated using the 2^−ΔΔCt^ method with β‐actin as an internal reference. Each experiment was conducted with three replicates. The relevant primers (Table 1) were designed by Sangon Biotechnology.

**TABLE 1:** Primer sequences.

Gene	Sequence (5′–3′)
LINC00470‐F	CGTAAGGTGACGAGGAGCTG
LINC00470‐R	GGGGAATGGCTTTTGGGTCA
GATAD1‐F	GGGCCTGAAGCCCACCTGCAGC
GATAD1‐R	TTGCCGCCCCCGCCCCCGTTG
TEAD4‐F	CACCATTACCTCCAACGAGTGGA
TEAD4‐R	AGCTTGATGTAGCGGGCAATCAG
IRF‐1‐F	ATGCCCATCACTCGGATGCGCA
IRF‐1‐R	TGGCCTTCCACGTCTTGGGATC
EGR2‐F	AGCTGTCTGACAACATCTACCC
EGR2‐R	CCATGTAAGTGAAGGTCTGGTTT
SOX4‐F	CGCAGATCGAGCGGCGCAAGATC
SOX4‐R	CCCACCGCCCGCGCCGCCCG
β‐Actin‐F	CTCGTCGTCGACAACGGCTCC
β‐Actin‐R	TTTTCTCCATGTCGTCCCAGTT

Abbreviations: EGR2, early growth response 2; F, forward; GATAD1, GATA zinc finger domain containing 1; IRF, interferon regulatory factor; R, reverse; SOX4, SRY‐related high‐mobility‐group box 4; TEAD4, TEAD4, TEA domain family member 4.

### 2.7 Western blot

LN229 and U87 cells were lysed with enhanced radioimmunoprecipitation assay (RIPA) lysis solution encompassing protease inhibitors (BOSTER) to obtain proteins, followed by protein concentration determination using the bicinchoninic acid protein quantification kit (BOSTER). After protein separation using 10% sodium dodecyl sulfate polyacrylamide gel electrophoresis, the separated proteins were electrotransferred onto polyvinylidene fluoride membranes. Subsequent to 2‐h blocking with 5% bovine serum albumin (BSA) at room temperature to block nonspecific binding, the membranes were probed overnight with diluted primary antibodies (Abcam) against GATA zinc finger domain containing 1 (GATAD1; ab181544, 1:10,000), TEA domain family member 4 (TEAD4; ab58310, 1:1000), interferon regulatory factor 1 (IRF1; ab243895, 1:1000), EGR2 (ab108399, 1:5000), SOX4 (ab70598, 1:1000), cyclinD1 (ab16663, 1:200), cyclin‐dependent kinase 4 (CDK4, ab108357, 1:5000), vimentin (ab92547, 1:1000), fibronectin (ab2413, 1:1000), LC3B (ab192890, 1:1000), and β‐actin (ab8226, 1:2500) at 4°C. After washing, membranes were incubated for 1 h at room temperature with horseradish peroxidase‐labeled secondary antibody and then incubated for 1 min at room temperature with electrogenerated chemiluminescence (ECL) working solution (EMD Millipore). Following the removal of the excess ECL reagent, membranes were sealed with cling film and developed and fixed by 5–10 min of exposure to X‐ray film in the dark box. The grayscale quantification of bands in western blot images was performed using the Image J analysis software (National Institutes of Health) with β‐actin as an internal reference. The experiment was repeated 3 times each.

### 2.8 Chromatin immunoprecipitation assay

After treatment of glioma cells with 4% formaldehyde (the final concentration of 1%), the cells were broken by ultrasonication and incubated with EGR2 antibodies (MA5‐38053, 1:200, Thermo Fisher Scientific) overnight at 4°C for binding to EGR2–SOX4 promoter complexes. Following the supplementation of protein A agarose/SaLmon sperm DNA to bind to the EGR2 antibody–EGR2–SOX4 promoter complex, the final complex was precipitated and washed to remove some nonspecific binding. After elution, the enriched EGR2–SOX4 promoter complex was harvested and centrifuged (12,000 *g*, 5 min) and the supernatant was discarded. The nonspecific complex was washed, decrosslinked overnight at 65°C, and purified by phenol/chloroform extraction for the recovery of DNA fragments. The enriched SOX4 promoter fragments were purified for PCR analysis. Immunoglobulin G (IgG, ab172730, 1:100, Abcam) served as a negative control (NC). The experiment was replicated 3 times.

### 2.9 Dual‐luciferase reporter assay

Artificially synthesized SOX4 promoter sequence fragments were inserted into the pMIR‐reporter (Promega) after enzyme digestion. Next, the correctly sequenced luciferase reporter plasmids were respectively cotransfected with LV‐NC, LV‐LIN00470, LV‐EGR2, si‐NC, and si‐LINC00470 into U87 cells. Following 48 h of transfection, the cells were lysed and centrifuged for 3–5 min, and the supernatant was obtained and analyzed for luciferase activity in the cell extracts using the dual‐luciferase reporter assay system (Promega). The luciferase activity was measured on a fluorescence detector (Promega) with the ratio of the target luciferase activity to the internal reference luciferase activity as the relative luciferase activity. The parallel experiment was repeated 3 times.

The three most likely binding sites of EGR2 protein to SOX4 DNA were identified through the JASPAR (http://jaspar.genereg.net/) website. Subsequently, recombinant luciferase reporter vectors of truncated or mutant binding sites were constructed and cotransfected with the EGR2 expression vector into U87 cells for a dual luciferase reporter assay to verify the specific site of EGR2 protein binding to SOX4 DNA. The exact method steps were the same as mentioned earlier.

### 2.10 RNA immunoprecipitation assay

The binding of LIN00470 to EGR2 was tested using the RNA immunoprecipitation (RIP) kit (EMD Millipore). Cells were washed with precooled PBS, followed by discarding of the supernatant. Then, cells were lysed with an equal volume of the RIPA lysis solution in an ice bath for 5 min and centrifuged (4°C, 21,000 *g*, 10 min) to acquire the supernatant. Part of the supernatant was utilized as input and part was incubated with antibody for coprecipitation. Specifically, for each coprecipitation reaction system, 50 μL magnetic beads were washed, resuspended in 100 μL RIP wash buffer, and incubated with 5 μg antibodies for binding according to the experimental groups. The magnetic bead–antibody complexes were washed, resuspended in 900 μL RIP wash buffer, and supplemented with 100 μL supernatant for overnight incubation at 4°C. The samples were washed 3 times and placed on magnetic holders to attain the magnetic bead–protein complexes. The samples and the input were digested by proteinase K to extract RNA for subsequent quantitative reverse real‐time polymerase chain reaction (qRT‐PCR) detection of LINC00470 expression. The antibodies adopted for RIP were as follows: EGR2 (1:200, MA5‐38053, Thermo Fisher Scientific) were mixed at room temperature for 30 min, and rabbit anti‐human IgG (1:100, ab172730, Abcam) functioned as the NC.

### 2.11 RNA pull‐down

Biotinylated LIN00470 and U6 RNA were mixed with proteins from nuclear extracts of cancer cells. The biotinylated LIN00470–protein complex was purified using streptavidin‐agarose beads (Thermo Fisher Scientific). Proteins were then eluted from the RNA–protein complex for immunoblotting using EGR2 antibodies.

### 2.12 Cell cycle analysis

The cultured glioma cells were centrifuged at 250 *g* for 5 min, washed with prechilled PBS, and fixed overnight at −20°C with 70% ethanol. The fixed cells were washed with PBS for 10 min, treated with RNAase A for 30 min, and then incubated with propidium iodide at room temperature for 30 min. BD FACSCalibur™ Flow Cytometer (BD Biosciences) was adopted to assess the cell cycle of each specific sample following the manufacturer's protocols. The experiment was replicated 3 times.

### 2.13 Transwell assay

Glioma cells were starved in serum‐free medium for 24 h, digested, rinsed twice with PBS, and resuspended in serum‐free DMEM containing 10 g/L BSA (Sigma‐Aldrich) to adjust the cell density to 3 × 10^4^ cells/mL. Experiments were performed using Transwell chambers (Corning; 8 μm) with 24‐well plates. Thereafter, 50 μL Matrigel matrix gel (Sigma‐Aldrich) were spread or not spread in the chambers before the experiments. Each group was set with three chambers. Each chamber was added with 100 μL cell suspension and the lower chambers were added with 600 μL of 10% L15 or Roswell Park Memorial Institute 1640 medium, followed by incubation at 37°C with 5% CO_2_. After 48 h, the chambers were fixed with 4% paraformaldehyde for 30 min, placed in 0.2% Triton X‐100 solution (Sigma‐Aldrich) for 15 min, and stained with 0.05% gentian violet for 5 min. Then, the stained cells in five random fields of view were counted under an inverted microscope (Leica DMi8 M/C/A; Leica Microsystems). The experiment was replicated 3 times.

### 2.14 Matrix gel‐based in vitro endothelial tube formation assay

As described in a prior study,^19^ endothelial cell tube formation was assessed using matrix gel‐coated slides. Experimental results were photographed using an Eclipse Ti microscope with a DS‐Fi1 camera (Nikon) at 40× magnifications, and the total area of endothelial cell‐formed vessels in each lumen was calculated using NIS‐Elements‐Basic Research software (Nikon) and presented as angiogenesis score. The experiment was replicated 3 times.

### 2.15 Flow cytometry for apoptosis analysis

Cells were centrifuged at 112 *g* for 10 min, followed by three PBS washes. The cell suspension was fixed with 70% ethanol (prechilled at −20°C) for 1 h and centrifuged to remove the fixing solution. Then, the cells were rinsed and mixed with PBS to make a single cell suspension of 1 × 10^7^ cells/mL. An Annexin V‐fluorescein isothiocyanate apoptosis assay kit (Beyotime) was applied for the detection of apoptosis, and a FACSCanto II flow cytometer system for the calculation of apoptosis percentage. Finally, the FlowJo 7.6.1 Software (BD Biosciences) was utilized for the analysis of flow cytometry results. The experiment was replicated 3 times.

### 2.16 Transmission electron microscopy

Correspondingly treated LN229 and U87 cells were digested with 0.25% trypsin containing EDTA, washed with PBS, and prefixed with 2.5% glutaraldehyde dissolved in a final concentration of 0.1 M sodium cacodylate buffer overnight at 4°C. After fixation, cells were treated with osmium tetroxide, followed by dehydration and embedding. Ultrathin sections (100 nm) were cut using an ultramicrotome (LEICA ULTRACUT UCT) and then stained with uranyl acetate and lead citrate, followed by examination with a Philips CM100 Transmission Electron Microscope. The experiment was replicated 3 times.

### 2.17 Tumor xenograft in nude mice

Thirty specific pathogen‐free (SPF) grade male BALB/c‐nu nude mice (aged 5–6 weeks, weighing 16–18 g, SLAC Laboratory Animal Central) were raised in a SPF‐grade sterile laminar flow room at the constant temperature of 22–26°C with the constant humidity of 55 ± 5%. U87 cells (1 × 10^6^) without any treatment or transfected with si‐LINC00470, LV‐LINC00470, or LV‐SOX4 were inoculated in the right anterior inguinal region of the mice. The tumor size was measured using calipers, and the tumor volume was calculated following the formula: (L × W^2^)/2, where L represented the length of the tumor and W represented the width of the tumor. When the mean tumor volume of nude mice reached approximately 100 mm^3^, mice and control mice were injected intraperitoneally with temozolomide (12.5 mg/kg) and equal amounts of saline, respectively. At the indicated time, tumor volumes were measured and mice were euthanized for following experiments.

### 2.18 An intracranial in situ graft tumor model with glioma cells

GL261 + luc cells were transfected with si‐LINC00470, treated with temozolomide, or transfected with LV‐LINC00470 or LV‐SOX4 and treated with temozolomide. Afterward, the logarithmically growing GL261 + luc cells were collected, and the cell density was adjusted to 1 × 10^3^ cells/μL. After anesthesia with sodium pentobarbital, male BALB/c mice (aged 4–6 weeks; Cyagen) were subjected to an intracranial injection with 5 μL of the cell suspension (5 × 10^3^ cells/mouse) with a protein microinjector. The injection site was 0.5 cm away from the right posterior side of the intersection of the anterior midline and lateral canthus of the mice, with the depth of injection as 0.5 cm. Dependent on the transfected GL261 + luc cells, the mice were correspondingly assigned into five groups (six mice per group): control, temozolomide, temozolomide + si‐LINC00470, temozolomide + LV‐LINC00470, and temozolomide + LV‐SOX4 groups. On the days 14 and 28 of graft tumor establishment, each mouse was injected with 200 μL luciferin substrate intraperitoneally. Ten minutes later, the tumor formation and luciferase signal intensity in mice were determined by the in vivo imaging system.

### 2.19 TdT‐mediated dUTP‐biotin nick end‐labeling staining

After completion of all the aforementioned animal experiments, the mice were euthanized under deep anesthesia and the brains were carefully extracted. The brain tissues were fixed in 4% paraformaldehyde and cryoprotected, followed by cutting of coronal sections using a cryosectioner (Slee Technik). The TdT‐mediated dUTP‐biotin nick end‐labeling (TUNEL) apoptosis assay kit (Thermo Fisher Scientific) was utilized to detect apoptosis as per the manufacturer's manuals. TUNEL‐positive cells in three microscopic fields (20×) from three independent coronal sections were imaged. The total number of cells in the captured images were counted and normalized with Abercrombie's correction factor. The mean percentage of positive cells was acquired from all images.

### 2.20 Statistical analysis

GraphPad prism8 software was applied for statistical analysis, and all data were summarized as mean ± standard deviation. The normal distribution of data was assessed with the Shapiro–Wilk test, and all data conformed to normal distribution. After normal distribution test, the *T* test was performed for comparisons between the two groups, and the one‐way analysis of variance test was applied for comparisons among multiple groups, with Tukey's multiple comparisons test for post hoc multiple comparisons, except for the results whose specific methods of analysis were described. The *p*_s_ < 0.05 were considered a statistically significant difference.

## 3 RESULTS

### 3.1 Temozolomide restrained glioblastoma cell survival through inhibition of LINC00470‐regulated EGR2

**FIGURE 1:**
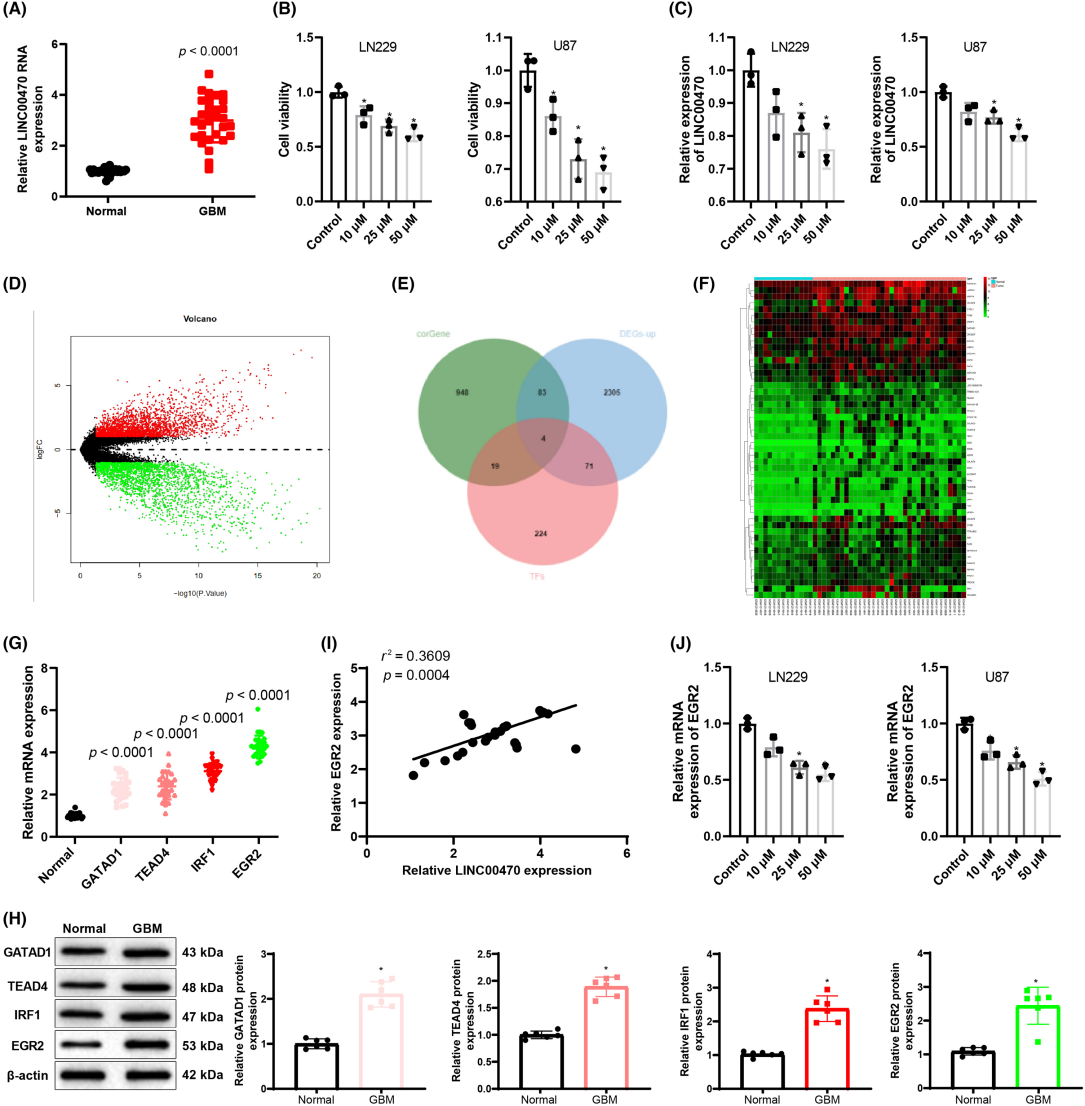
Temozolomide inhibits LINC00470‐regulated EGR2 and thus represses glioblastoma cell survival. (A) LINC00470 expression in normal brain tissues and brain tissues of glioblastoma patients was measured by qRT‐PCR. (B) Cell viability of glioma cell lines LN229 and U87 after temozolomide treatment was examined with CCK‐8 assay. (C) LINC00470 expression in LN229 and U87 cells after temozolomide treatment were evaluated by qRT‐PCR. (D) The volcano plot of differential genes in glioma‐related microarray GSE50161 was presented, in which the horizontal coordinates indicated ‐log10 *p* value, the vertical coordinates indicated logFC, red dots indicated substantially highly expressed genes in the tumor, and green dots indicated prominently lowly expressed genes in the tumor. (E) The downstream regulatory transcription factors of LINC00470 were predicted. The three circles in the figure indicated LINC00470 coexpressed genes, considerably upregulated genes, and transcription factor data in microarray GSE50161, respectively, and the middle part indicated the intersection of the three sets of data. (F) The heat map of expression of some dramatically upregulated genes including candidate transcription factors was presented. Horizontal coordinates indicated sample numbers, and vertical coordinates indicated gene names, with color scale in the right histogram. (G) The mRNA expression of GATAD1, TEAD4, IRF1, and EGR2 in glioblastoma patient and normal brain tissue specimens was detected by qRT‐PCR (*n* = 30). (H) The protein expression of GATAD1, TEAD4, IRF1, and EGR2 in glioblastoma patient and normal brain tissue specimens was assessed by western blot (*n* = 30). (I) Correlation of LINC00470 and EGR2 mRNA expression in brain tissues of glioblastoma patients was analyzed by Pearson's correlation coefficient analysis. (J) EGR2 mRNA expression in LN229 and U87 cells after temozolomide treatment were evaluated by qRT‐PCR. *p < 0.05, compared with the control group. Data were derived from three independent replicate experiments and compared using one‐way analysis of variance with Tukey's test for post hoc multiple comparisons. EGR2, early growth response 2; IRF1, interferon regulatory factor 1; LV, lentiviral vectors for overexpression; si, small interfering RNA; TEAD4, TEA domain family member 4; TMZ, temozolomide.

Quantitative reverse transcription polymerase chain reaction (qRT‐PCR) results of brain tissue samples attained from clinical patients with glioblastoma exhibited that the brain tissues of patient with glioblastoma had highly expressed LINC00470 (Figure 1A). Also, our results demonstrated that temozolomide treatment dramatically decreased cell viability and LINC00470 expression in glioblastoma cells (Figure 1B,C).

To further understand the regulatory mechanism of LINC00470, an analysis was first conducted on the glioma‐related expression microarray GSE50161 obtained from the Gene Expression Omnibus database, which yielded 2463 genes prominently highly expressed in gliomas (Figure 1D). The coexpressed genes of LINC00470 in glioma data of The Cancer Genome Atlas were further searched and intersected with the highly expressed genes in the GSE50161 microarray and the transcription factor data downloaded from the Cistrome database (Figure 1E,F). Four candidate transcription factors (GATAD1, TEAD4, IRF1, and EGR2) were finally obtained and were found to be considerably highly expressed in specimens of patients with glioblastoma according to the results of qRT‐PCR and western blot (Figure 1G,H). LINC00470 and EGR2 expression in specimens of patients with glioblastoma were measured using qRT‐PCR, and a remarkable positive correlation between LINC00470 and EGR2 was observed by the Pearson's correlation coefficient analysis (Figure 1I), which suggested that LINC00470 most likely exerted regulatory functions through EGR2. Follow‐up qRT‐PCR experiments also displayed that EGR2 mRNA expression in temozolomide‐treated LN229 and U87 cells gradually decreased with increasing temozolomide concentration (Figure 1J). Moreover, the results in Figure 1C also described that LINC00470 expression in temozolomide‐treated LN229 and U87 cells decreased progressively with elevating temozolomide concentration (subsequent experiments were performed using 25 μM temozolomide). Collectively, temozolomide might affect glioblastoma survival by suppressing LINC00470‐modulated EGR2.

### 3.2 LINC00470 transcriptionally activated SOX4 through EGR2

**FIGURE 2:**
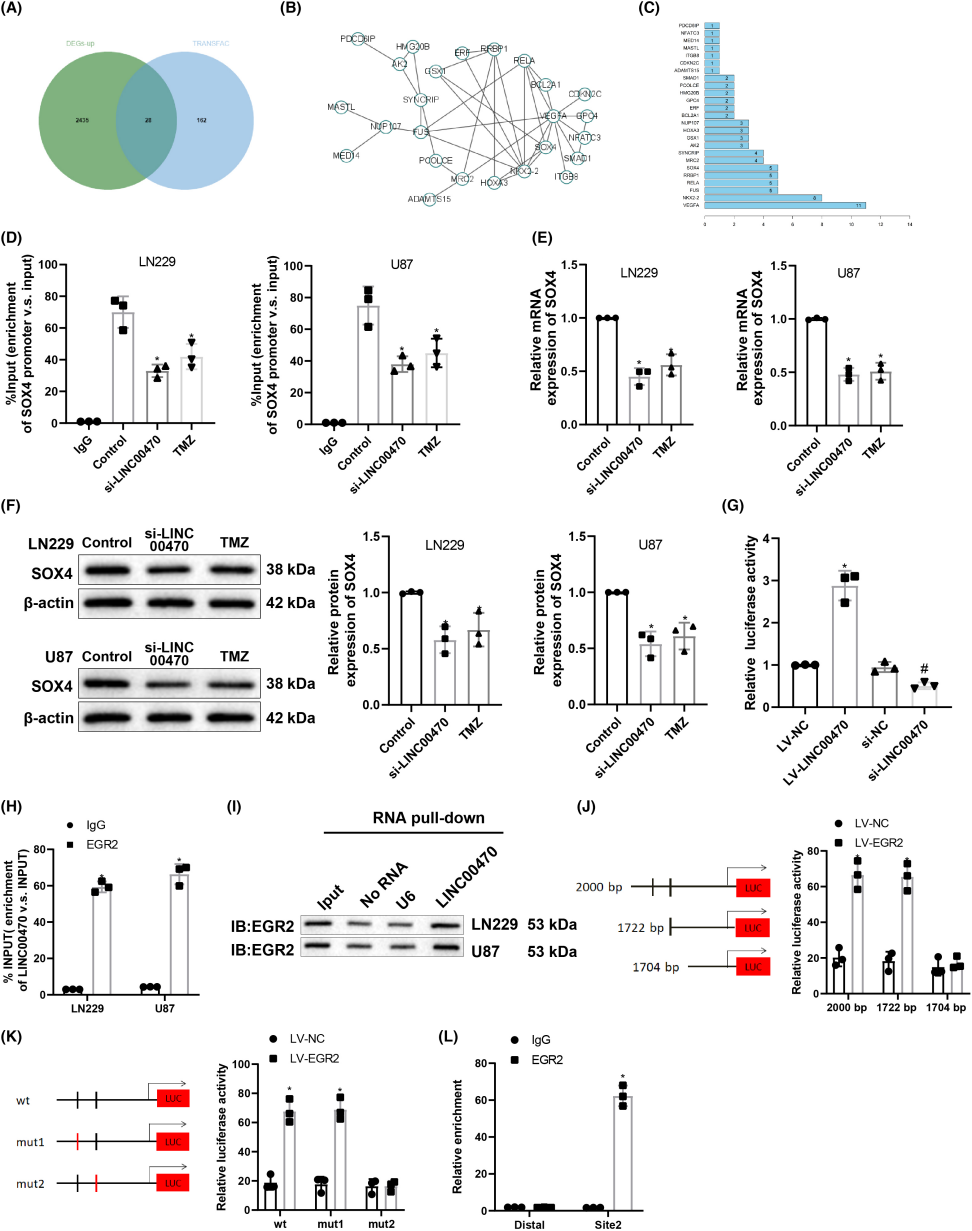
LINC00470 increases SOX4 expression via EGR2. (A) Intersection of prominently upregulated genes in GSE50161 and predicted results of target genes of EGR2 was exhibited. (B) Interaction analysis on candidate target genes of EGR2 was detailed, in which each circle represented a gene and the linkage between the circles indicated the existence of interaction between genes (the more genes linkage, the higher the degree value). (C) The degree value statistics of candidate target genes were displayed. The horizontal coordinate indicated the degree value and the vertical coordinate indicated the gene name. (D) SOX4 promoter enrichment in cells was measured by ChIP assay. (E) SOX4 mRNA expression in cells was detected by qRT‐PCR. (F) SOX4 protein expression in cells was evaluated by western blot. (G) The luciferase activity of SOX4 after overexpression or knockdown of LINC00470 was evaluated by dual‐luciferase reporter assay. (H) The association between LINC00470 and EGR2 was assessed by RIP assay. (I) The interaction of LINC00470 and EGR2 was determined by RNA pull‐down assay. (J) The relative luciferase activity after transfection with different lengths of SOX4 promoters and overexpression of EGR2 in U87 cells was tested by dual‐luciferase reporter assay. (K) The relative luciferase activity after transfection of wild and mutant SOX4 promoters and overexpression of EGR2 in U87 cells was examined by dual‐luciferase reporter assay. (L) SOX4 promoter enrichment in cells was assessed by ChIP assay. *p < 0.05, compared with the control, IgG, or LV‐NC group. ^#^ *p* < 0.05, compared with the si‐NC group. Data were derived from three independent replicate experiments and compared using one‐way analysis of variance with Tukey's test for post hoc multiple comparisons. EGR2, early growth response 2; LV, lentiviral vectors for overexpression; si, small interfering RNA; SOX4, SRY‐related high‐mobility‐group box 4; TMZ, temozolomide.

Bioinformatics analysis and experiments initially revealed that LINC00470 might exhibit regulatory effects in glioblastoma via EGR2 during temozolomide treatment. The downstream target genes of EGR2 were further predicted by TRANSFAC database and were intersected with the remarkably upregulated genes in GSE50161 (Figure 2A). Thus, 28 candidate target genes were attained and analyzed for gene interactions to construct a gene interaction network graph (Figure 2B), followed by counting of the degree value of each gene (Figure 2C). The results demonstrated that six genes, including SOX4, were in a relatively core position in the gene interaction network graph, and SOX4 had the largest upregulation fold in GSE50161 among these six genes (Table S2).

Next, chromatin immunoprecipitation (ChIP) assays presented that knockdown of LINC00740 in LN229 and U87 cells resulted in a striking reduction in SOX4 promoter enrichment, and the same trend was observed for SOX4 promoter enrichment in temozolomide‐treated cells (Figure 2D). As revealed in qRT‐PCR and western blot experiments, knockdown of LINC00470 or temozolomide treatment markedly diminished SOX4 expression in LN229 and U87 cells (Figure 2E,F). To confirm the regulation of LINC00470 on SOX4, SOX4 promoter sequence was cloned into pMIR reporter, and the luciferase activity was assessed by the dual‐luciferase reporter assay after cotransfection of pMIR‐reporter plasmid with si‐LINC00470 or LV‐LINC00470 into U87 cells. The results displayed that overexpression of LINC00470 appreciably enhanced but knockdown of LINC00470 evidently decreased luciferase activity (Figure 2G).

The relationship between LINC00470 and EGR2 was further clarified by RIP experiments, which demonstrated that the EGR2 antibody enriched more LINC00470 than the IgG antibody (Figure 2H). Further RNA‐pulldown assay also exhibited that LINC00470 specifically bound to EGR2 (Figure 2I).

Meanwhile, the presence of the EGR2 binding structural domain in the SOX4 promoter region (nt: 1704–1718; 1722–1736) was identified by the JASPAR database (Table S3). Then, the truncated or mutant SOX4 recombinant luciferase reporter gene vectors were cotransfected with EGR2 expression vector into U87 cells for the dual luciferase reporter assay (Figure 2J,K), which manifested no substantial change of luciferase activity in mutant or truncated binding site 2 (motif: TATACACACACACACGGAC)‐treated U87 cells, indicating that the motif 2 was the binding site between EGR2 and SOX4. The binding of EGR2 at the site 2 in the SOX4 promoter region was further tested by ChIP assay (Figure 2L). The results exhibited that the amount of amplification products obtained from site 2 primers was observably higher than that obtained from the distal primers in the EGR2 group (*p* < 0.05), while there was no evident difference in the amount of amplification products between the two pairs of primers in the IgG group (*p* > 0.05), indicating that the site 2 in the SOX4 promoter region was a specific binding site for EGR2. These results illustrated that LINC00470 specifically bound to EGR2 to activate downstream SOX4 expression.

### 3.3 Temozolomide restricted cell proliferation, migration, invasion, and angiogenesis in glioblastoma by inhibiting the LINC00470/EGR2/SOX4 axis

**FIGURE 3:**
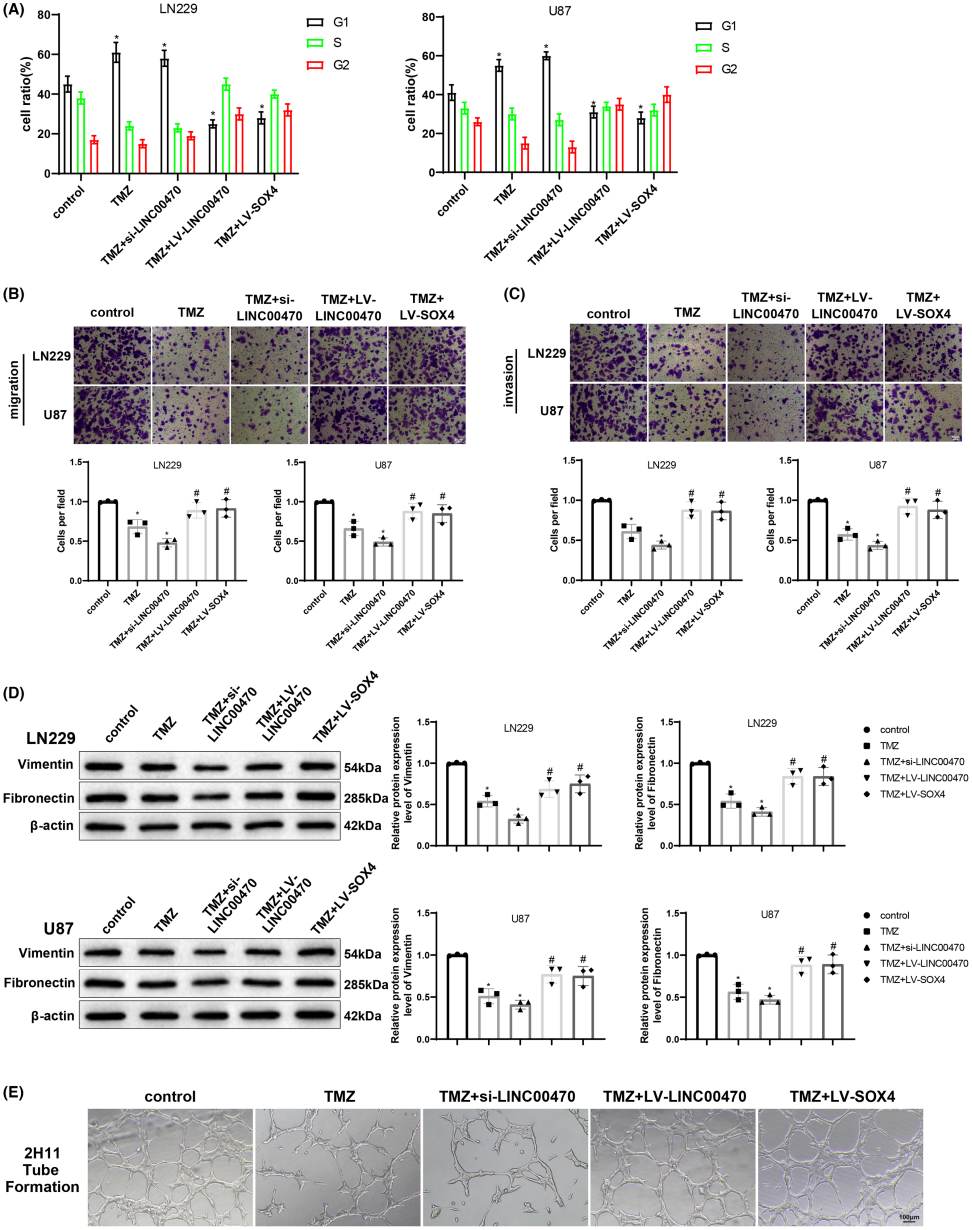
Temozolomide treatment restrains the LINC00470/EGR2/SOX4 axis and thereby inhibits cell proliferation, migration, invasion, and angiogenesis in glioblastoma. (A) Cycle distribution of LN229 and U87 cells was examined by flow cytometry. (B) The migration ability of cells was detected by Transwell assay. (C) The invasion ability of cells was detected by Transwell assay. (D) The protein expression of vimentin and fibronectin in cells was evaluated by western blot. (E) Angiogenic capacity was examined by matrix gel‐based in vitro endothelial tube formation assay using 2H11 cells with different treatments. *p < 0.05, compared with the control group; ^#^ *p* < 0.05, compared with the TMZ group. Data were derived from three independent replicate experiments and compared using one‐way analysis of variance with Tukey's test for post hoc multiple comparisons. EGR2, early growth response 2; LV, lentiviral vectors for overexpression; si, small interfering RNA; SOX4, SRY‐related high‐mobility‐group box 4; TMZ, temozolomide.

To further identify whether the LINC00470/EGR2/SOX4 axis assumed an essential role in the effects of temozolomide treatment on glioblastoma, cell cycle distribution under different treatments was first examined by flow cytometry. The results demonstrated that in LN229 and U87 cells, temozolomide treatment or cotreatment with temozolomide + si‐LINC00470 obviously augmented the number of G1 phase cells, while an opposite trend was found in cells treated with temozolomide + LV‐LINC00470 or temozolomide + LV‐SOX4 compared with cells treated with temozolomide (Figure 3A). As reflected by Transwell assay results, overexpression of LINC00470 or SOX4 restored the reduced migratory and invasive properties of LN229 and U87 cells induced by temozolomide treatment to some extent (Figure 3B,C). Western blot results revealed that overexpression of LINC00470 or SOX4 nullified the decreased expression of mesenchymal surface marker proteins vimentin and fibronectin by temozolomide treatment to some extent (Figure 3D). In addition, LINC00470 or SOX4 overexpression also annulled temozolomide‐diminished angiogenesis (Figure 3E). In addition, it was also found that combination of temozolomide treatment with LINC00470 knockdown was more effective than temozolomide treatment alone regarding the above indices. Meanwhile, LN229 and U87 cells were treated with temozolomide and transfected with si‐LINC00470 and LV‐EGR2, followed by flow cytometry, Transwell, western blot, and tube formation assays. The results presented that LV‐EGR2 transfection strikingly reduced the number of G1 phase cells (Figure S1A), enhanced cell migratory and invasive capabilities (Figure S1B,C), elevated vimentin and fibronectin expression in cells (Figure S1D), and augmented cell angiogenesis (Figure S1E) in the presence of treatment with temozolomide + si‐LINC00470. Collectively, temozolomide might repress cell proliferation, migration, invasion, and angiogenesis in glioblastoma through the disruption of the LINC00470/EGR2/SOX4 axis.

### 3.4 Temozolomide subdued glioblastoma growth in vivo by inactivating the LINC00470/EGR2/SOX4 axis

**FIGURE 4:**
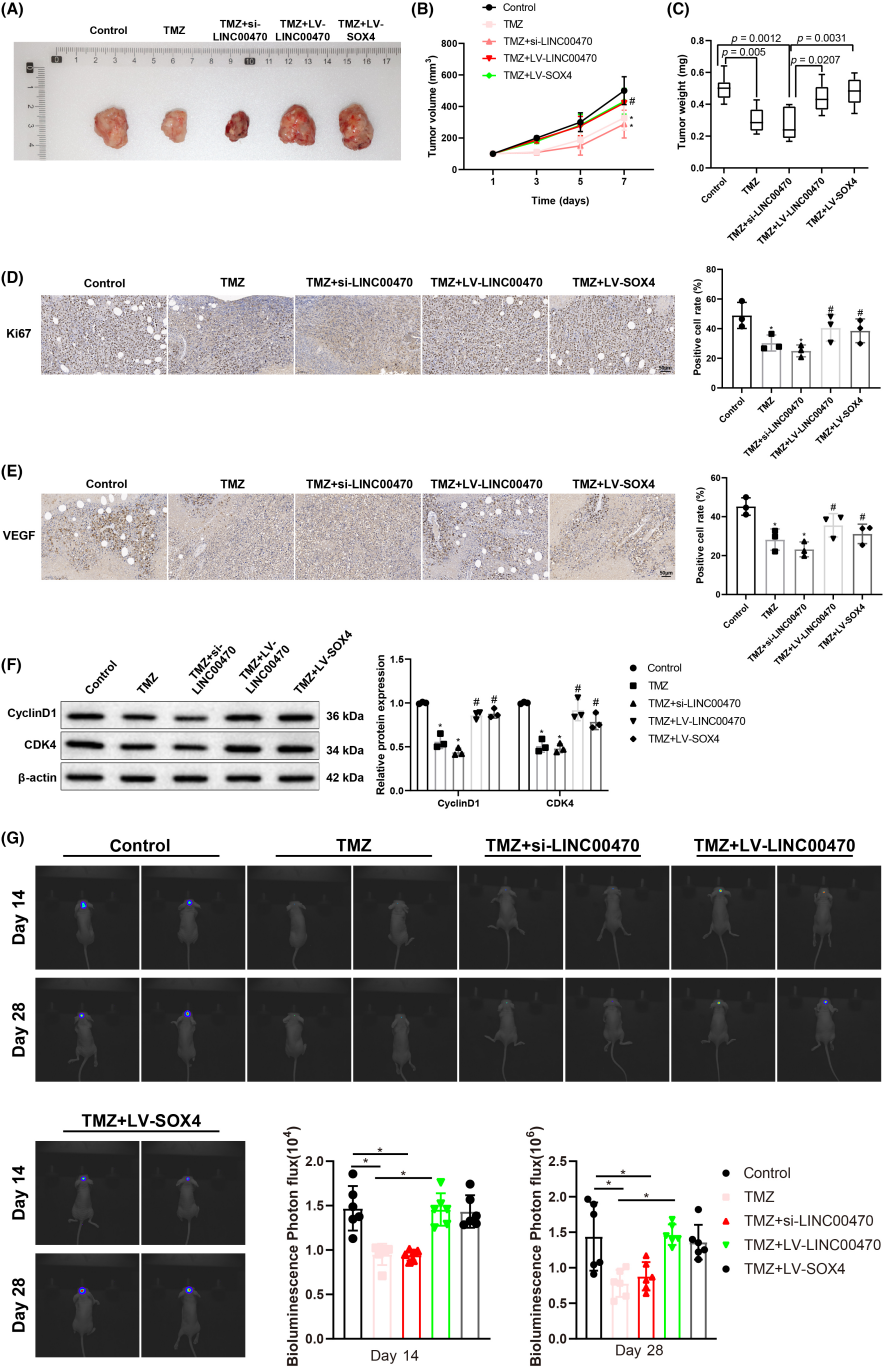
Temozolomide treatment delays glioblastoma growth in nude mice via the inhibition of the LINC00470/EGR2/SOX4 axis. (A) Tumors extracted from mice were photographed. (B) Tumor volumes were measured at the times shown in the figure, *n* = 6 mice in each group; *p < 0.05, compared with the control group, ^#^ *p* < 0.05, compared with the TMZ group; and data were analyzed by two‐way analysis of variance with LSD test for post hoc multiple comparisons. (C) Tumors from each group of nude mice were weighed. (D) Ki‐67 expression in the tumor tissues of nude mice was measured by IHC. (E) VEGF expression in the tumor tissues of nude mice was evaluated by IHC. (F) The protein expression of cyclinD1 and CDK4 in nude mice was examined by western blot (data represent three independent replicate trials). (G) Tumor size in mice of intracranial in situ graft tumor model was determined by in vivo animal imaging, *n* = 6; *p < 0.05, compared with the control group; ^#^ *p* < 0.05, compared with the TMZ group. Except where noted, other data were analyzed using one‐way analysis of variance with Tukey's test for post hoc multiple comparisons. CDK4, cyclin‐dependent kinase 4; EGR2, early growth response 2; IHC, immunohistochemistry; LSD, least significant difference; LV, lentiviral plasmids for overexpression; si, small interfering RNA; SOX4, SRY‐related high‐mobility‐group box 4; TMZ, temozolomide; VEGF, vascular endothelial growth factor.

To fully exploit whether temozolomide also acts on tumors in vivo via the LINC00470/EGR2/SOX4 axis, a subcutaneous glioblastoma model was established by injection of U87 cells into nude mice. As presented in Figure 4A–C, temozolomide treatment or the combination of temozolomide treatment and knockdown of LINC00470 prominently diminished the volume and weight of tumors in nude mice. Overexpression of LINC00470 or SOX4 partially abolished the suppressive effect of temozolomide and conspicuously elevated tumor volume and weight in nude mice in the presence of temozolomide. The results of IHC experiments highlighted the remarkable diminishment in Ki67 and VEGF expression in tumor tissues caused by temozolomide treatment or the combination of temozolomide treatment with LINC00470 knockdown. In addition, the repressive effects of temozolomide treatment on Ki67 and VEGF expression in tumor tissues were partially rescued by the overexpression of LINC00470 or SOX4 (Figure 4D,E). As evidenced by western blot results, the protein expression of cyclinD1 and CDK4 was appreciably reduced in tumor tissues following temozolomide treatment or the simultaneous treatment of temozolomide and si‐LINC00470, while there were reverse trends in mice treated with temozolomide + LV‐LINC00470 or temozolomide + LV‐SOX4 versus mice treated with temozolomide (Figure 4F). The results of in vivo experiments were consistent with the results of in vitro experiments. Furthermore, an intracranial in situ graft tumor model was established with glioma cells to further confirm these results. In vivo imaging assays of mice on days 14 and 28 displayed that temozolomide treatment alone or simultaneous treatment of temozolomide and si‐LINC00470 substantially reduced tumor size in mice, while LV‐LINC00470 or LV‐SOX4 transfection markedly abolished the effect of temozolomide treatment in mice (Figure 4G). Overall, temozolomide treatment might block the LINC00470/EGR2/SOX4 axis to constrain glioblastoma growth in vivo.

### 3.5 Temozolomide treatment promoted apoptosis and autophagy of glioblastoma cells by inhibiting the LINC00470/EGR2/SOX4 axis in vivo and in vitro

**FIGURE 5:**
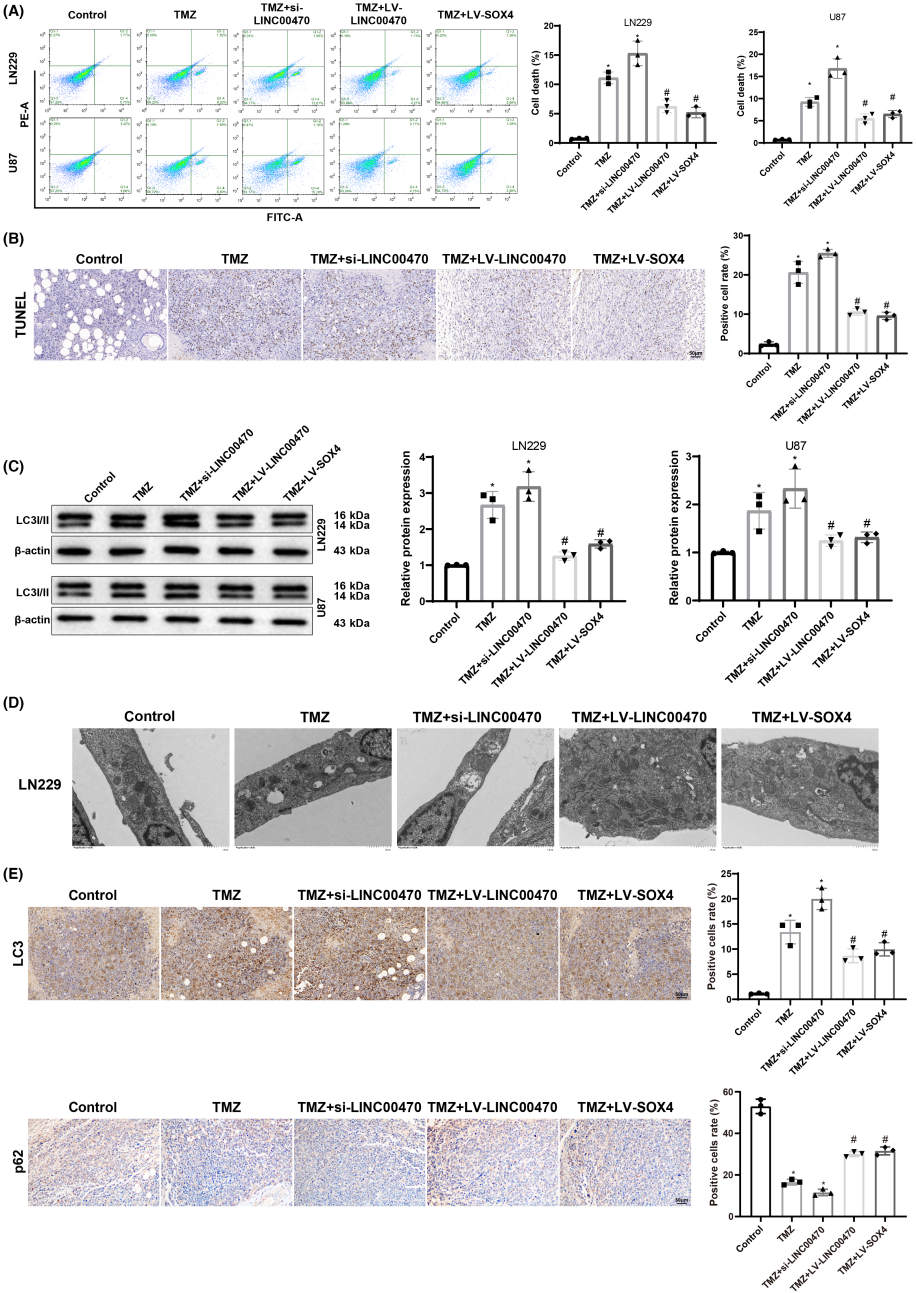
Temozolomide treatment boosts cell apoptosis and autophagy in glioblastoma by blocking the LINC00470/EGR2/SOX4 axis. (A) Cell apoptosis was measured by flow cytometry. (B) Apoptosis rate of tumor tissues from nude mice was evaluated by TUNEL (*n* = 6). (C) LC3II protein expression was detected by western blot in LN229 and U87 cells. (D) Cell autophagy was assessed by transmission electron microscopy. (E) LC3 and p62 expression in tumor tissues of nude mice was determined by IHC. *p < 0.05, compared with the control group; ^#^ *p* < 0.05, compared with the TMZ group. Cell experiments were repeated 3 times, and data were compared using one‐way analysis of variance with Tukey's test for post hoc multiple comparisons. EGR2, early growth response 2; IHC, immunohistochemistry; LV, lentiviral plasmids for overexpression; si, small interfering RNA; SOX4, SRY‐related high‐mobility‐group box 4; TMZ, temozolomide.

This study further ascertained whether the regulation of temozolomide on cell apoptosis and autophagy in glioblastoma also involved the LINC00470/EGR2/SOX4 axis. First, flow cytometry demonstrated that the apoptosis rate was considerably elevated by temozolomide treatment or the simultaneous treatment of temozolomide and si‐LINC00470, but conspicuously diminished in LN229 and U87 cells treated with temozolomide + LV‐LINC00470 or temozolomide + LV‐SOX4 in comparison to cells treated with temozolomide (Figure 5A). With respect to TUNEL results, the positive cell rate was observably elevated after temozolomide treatment or the combination of temozolomide treatment and LINC00470 knockdown, while the opposite trend was noticed after the treatment with temozolomide + LV‐LINC00470 or temozolomide + LV‐SOX4 versus temozolomide treatment (Figure 5B). This was concordant with the results of the aforementioned in vitro experiments. As depicted in western blot results, LC3II protein expression was dramatically elevated by temozolomide treatment or treatment with temozolomide + si‐LINC00470, but treatment with temozolomide + LV‐LINC00470 or temozolomide + LV‐SOX4 markedly lowered LC3II protein expression compared with temozolomide treatment (Figure 5C). TEM observations displayed a notable augmentation of autophagic vesicles following treatment with temozolomide or temozolomide + si‐LINC00470 compared to control treatment, but a prominent reduction of autophagic vesicles subsequent to temozolomide treatment combined with the overexpression of LINC00470 or SOX4 versus temozolomide treatment (Figure 5D). In addition, IHC results depicted the enhanced rate of LC3‐positive cells and the reduced p62 positive expression induced by temozolomide treatment or temozolomide treatment with concomitant knockdown of LINC00470. Additionally, these impacts of temozolomide treatment was partially neutralized by the overexpression of LINC00470 or SOX4 (Figure 5E). Altogether, temozolomide treatment might also facilitate apoptosis and autophagy of glioblastoma cells in vivo and in vitro by blocking the LINC00470/EGR2/SOX4 axis.

## 4 DISCUSSION

Glioblastoma, a heterogeneous tumor, is defined as inherited and epigenetic mutations in tumor cells, making therapeutic approaches to eliminate all tumor cells a challenge and not yet possible.^20^ Regardless of numerous clinical trials, standard therapy for more than a decade remains surgical excision, radiotherapy, and temozolomide chemotherapy.^21^ However, full surgery excision is elusive due to the rapid proliferative capacity and high infiltrative growing of glioblastoma, while temozolomide chemotherapy can prolong the postsurgical survival of patients.^22^ Therefore, it is essential and meaningful to figure out detailed molecular mechanisms of temozolomide therapy in glioblastoma with the aim to enhance the therapeutic effect of temozolomide. This study illustrated that temozolomide suppressed cell proliferation, migration, invasion, and angiogenesis, but accelerated cell apoptosis, autophagy in glioblastoma, as well as restricted tumor growth in vivo, by disrupting the LINC00470/EGR2/SOX4 axis.

Temozolomide, a type of alkylating drug authorized for glioblastoma anticancer therapy, is able to cross the blood–brain barrier damaging DNA and initiating cellular suicide.^23^ Mat et al. observed that temozolomide treatment could repress cell cycle arrest in G2/M cells in glioblastoma.^24^ A number of previous articles proposed that temozolomide treatment could induce apoptosis and autophagy of glioblastoma cells.^25^, ^26^ Experimental data from Vengoji et al.^27^ demonstrated that temozolomide treatment suppressed migration, invasion, and proliferation of glioblastoma cells. Analogous to their article, our experimental results revealed that temozolomide treatment not only repressed the malignant behaviors (like cell proliferation, invasion, migration, and angiogenesis) and also facilitated cell apoptosis and autophagy in glioblastoma. As a hallmark of glioblastoma pathogenesis, angiogenesis is also an ongoing goal of glioblastoma treatment and intervention.^28^ Similarly, an earlier study found that temozolomide treatment reduced the vascular density and vascular endothelial growth factor expression (which are strongly associated with angiogenesis) in mice with glioblastoma.^29^ Although plenty of studies have focused on the molecular mechanisms underpinning temozolomide resistance and sensitivity in cancers,^30^, ^31^ the molecular mechanisms involved in temozolomide therapy have never been studied. Hence, the research of the molecular mechanisms behind temozolomide therapy in glioblastoma may bring novel ideas to enhance the efficiency of temozolomide therapy.

LINC00470 acted as oncogene to inhibit autophagy and facilitate chemoresistance in chronic myeloid leukemia.^32^ A prior study indicated that LINC00470 was highly expressed in the serum exosomes of glioblastoma patients.^33^ Intriguingly, high LINC00470 expression was also exhibited in brain tissues from patients with glioblastoma in our study. Findings in this study also revealed that temozolomide treatment prominently diminished LINC00470 expression in glioblastoma cells. Moreover, the research of Yan et al. manifested the accelerated the proliferation, migration, and invasion in gastric cancer cells after overexpressing LINC00470.^34^ Importantly, a prior article indicated that LINC00470 could boost invasion and proliferation of glioma cells and dampen chemosensitivity of temozolomide.^35^ Interestingly, a prior work proved that LINC00470 knockdown boosted the accumulation of autophagosomes in the cytoplasm of glioblastoma cells and enhanced the ratio of LC3II/LC3I.^36^ This study also manifested that the protective effect of temozolomide treatment on glioblastoma was further enhanced by LINC00470 knockdown but abolished by LINC00470 overexpression.

SOX4 assumes a carcinogenic role in prostate, bladder, and breast cancers by fostering cancer cell proliferation, migration, and invasion.^37^, ^38^, ^39^ SOX4 forms a complex of transcripts with OCT‐4 protein that triggers SOX2 expression and enhances the carcinogenicity of glioma cells.^40^ Mounting articles exhibited that SOX4 was highly expressed in glioma and glioblastoma tissues.^41^, ^42^ In this study, LINC00470 knockdown or temozolomide treatment both diminished SOX4 expression in glioblastoma cells. However, the association between LINC00470 and SOX4 has not been identified. Bioinformatics analysis in our research uncovered that both LINC00470 and SOX4 bound to EGR2, and then RIP, RNA pull‐down, ChIP, and dual‐luciferase reporter assays illustrated that LINC00470 activated the downstream transcription factor EGR2 to enhance SOX4 expression. In addition, our data also revealed that EGR2 and LINC00470 expression decreased with the elevation of temozolomide concentration in glioblastoma cells. EGR2, one of the zinc‐finger transcription factors belonging to the family of early growth response genes, has been discovered to facilitate cell invasion in glioma.^16^ SOX4 was implicated in the promotion of self‐renewal and stemness in gliomas.^43^ It was discovered in a prior work that the ALK pathway includes the SOX4, Sta3, Akt, and N‐myc activities, which jointly facilitated cell proliferation and tumor neovascularization in nonhypoxic contexts in glioblastoma.^44^ Intriguingly, our study identified that SOX4 overexpression also abrogated the therapeutic effect of temozolomide treatment on glioblastoma. Moreover, EGR2 overexpression nullified the inhibitory effects of temozolomide treatment and LINC00470 knockdown on glioblastoma cell malignant behaviors. Taken together, temozolomide may restrain the malignant behaviors of glioblastoma by dampening the LINC00470/EGR2/SOX4 axis. The effects of temozolomide via the LINC00470/EGR2/SOX4 axis on tumor growth as well as apoptosis and autophagy were also evidenced in nude mice.

**FIGURE 6:**
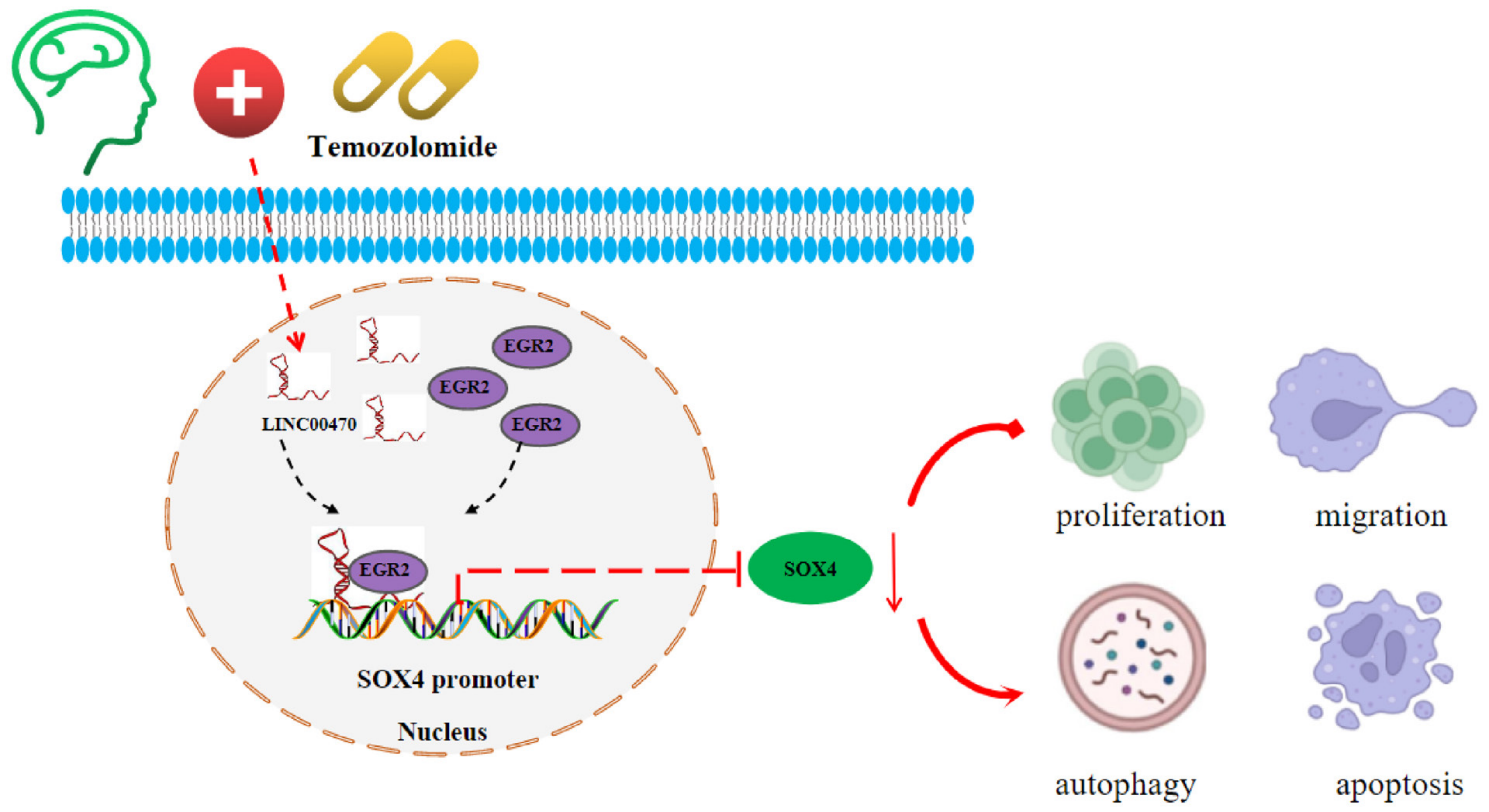
Molecular mechanism diagram. Temozolomide treatment suppressed glioblastoma progression by inhibiting LINC00470‐regulated EGR2 and thus decreasing SOX4 expression.

In conclusion, our research innovatively and first explored the molecular mechanism of temozolomide in glioblastoma. Specifically, temozolomide treatment restricted cell invasion, migration, and angiogenesis but facilitated cell autophagy and apoptosis in glioblastoma via the inactivation of the LINC00470/EGR2/SOX4 axis (Figure 6). However, this work is limited by the fact that the impacts of temozolomide via the LINC00470/EGR2/SOX4 axis on angiogenesis in vivo was not verified, which requires further research. This work intends to provide theoretical basis and fresh perspectives for glioblastoma management with temozolomide. Thus, further research in the future is warranted whether the combination of temozolomide with the inhibitors of small molecules studied here can improve the efficiency of temozolomide therapy.

## AUTHOR CONTRIBUTIONS

Mingming Zhang, Ping Liu, and Yan Cui conceived the ideas and designed the experiments. Wenyang Li, Ming Wang, and Jiarong He performed the experiments. Wenyang Li, Wenjia Ma, and Ming Wang analyzed the data and provided critical materials. Wenyang Li wrote the manuscript. Yan Cui supervised the study. All the authors have read and approved the final version for publication.

## FUNDING INFORMATION

This research was funded by grants from the Scientific Research Project of Health Commission of Hunan Province (No. 202104040152) and the Natural Science Foundation of Hunan Province (2022JJ30800).

## CONFLICT OF INTEREST STATEMENT

The authors declare that they have no competing interests.

## Supporting information


Table S1.

Figure S1.

Data S1.

Figure Caption

## Data Availability

The datasets used or analyzed during the current study are available from the corresponding author on reasonable request.
