# Corrigendum

**DOI:** 10.1111/cns.14317

**Published:** 2023-06-22

The authors accidently found the cell migration image in Control group in U87 cell line (figure 3B) was used mistakenly used for cell invasion in TMZ + LV‐SOX4 in U87 cell line (figure 3C). Therefore, the online image in figure 3B (control group in U87 cell line) shares the same view in the boundary with figure 3C (TMZ + LV‐SOX4 in U87 cell line). This mistake was made due to carelessness during image formatting using AI software. The authors have carefully checked the rest of the images and confirmed there is no other image issue that would influence the credibility of this research.

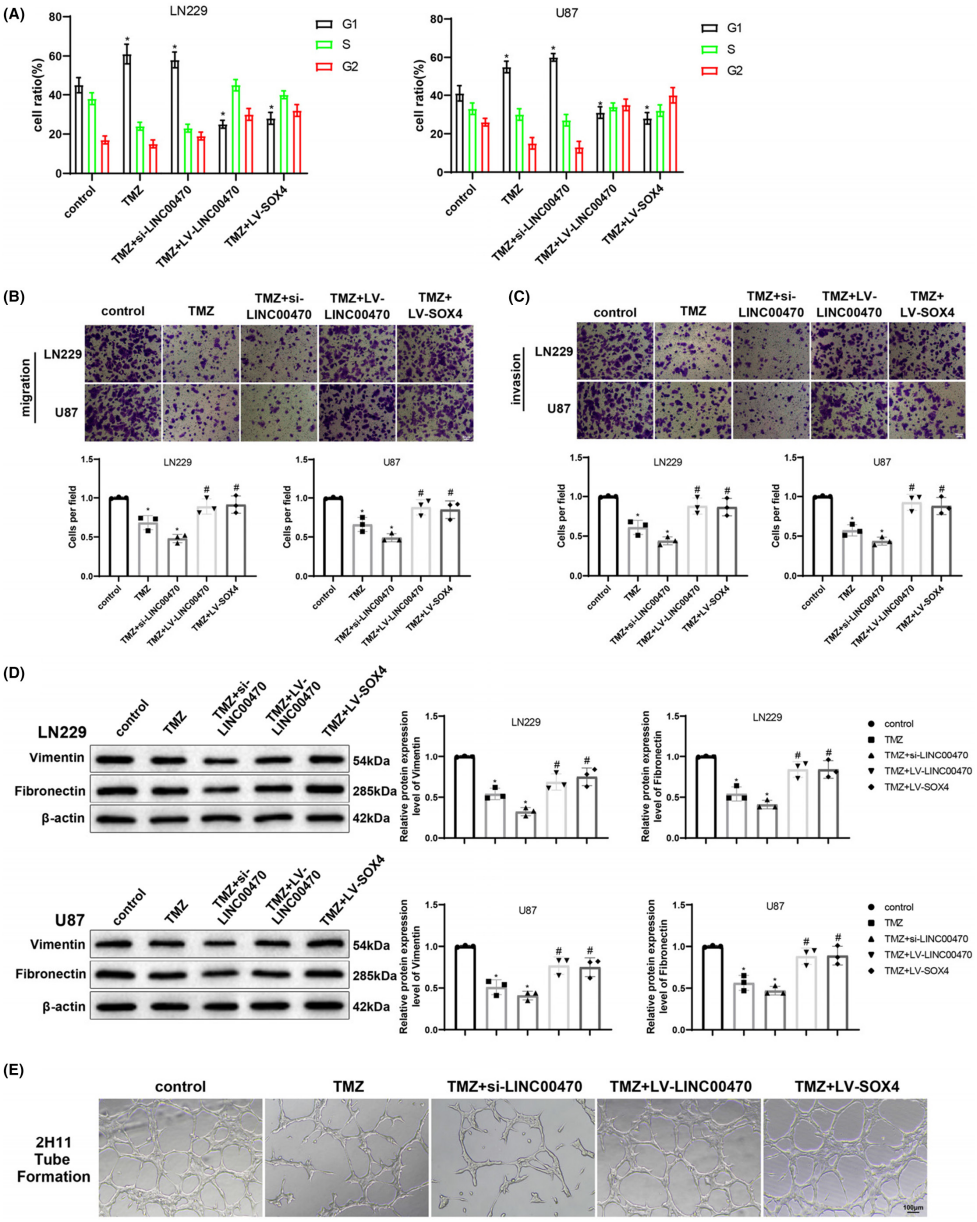

All listed authors agreed that those mistakenly used images need to be updated and believe updating the images will not show any effect on the results and conclusion of this article.The correct figure 3 and the raw images for migration and invasion below.

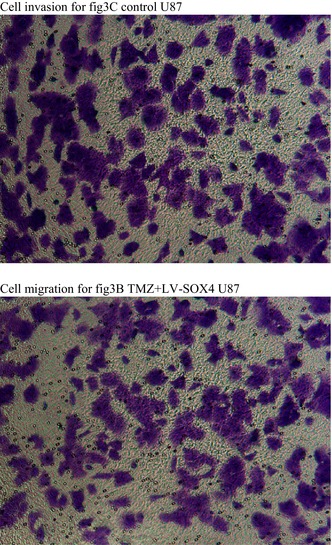

The Authors sincerely apologize for this error.

