# Supplementary material for: Temozolomide protects against the progression of glioblastoma via SOX4 downregulation by inhibiting the LINC00470‐mediated transcription factor EGR2
Source: CNS Neurosci Ther. 2023 Mar 29;29(8):2292–307. doi: 10.1111/cns.14181 (PMC10352878; doi:10.1111/cns.14181)
Supplement: Supplementary file 1 — Table S1. [file CNS-29-2292-s003.docx]

**Supplementary Table 1.** siRNA sequences

| gene | Sequence |
| --- | --- |
| si-LINC00470 | CACCGAGCTTATATTTGGTGTGTTT |
| si-NC | CACCGAATTTATGTTGTGGTCGTTT |

**Supplementary table 2.** Differential expression of candidate target genes

| symbol | logFC | *P*.Value | adj.*P*.Val |
| --- | --- | --- | --- |
| FUS | 1.094644422 | 2.93E-07 | 3.26E-06 |
| VEGFA | 2.205389287 | 1.01E-06 | 9.51E-06 |
| SOX4 | 2.219519556 | 4.04E-06 | 3.14E-05 |
| RELA | 1.109482189 | 3.13E-05 | 0.00018733 |
| RRBP1 | 1.068382535 | 0.000140762 | 0.000694071 |
| NKX2-2 | 2.192915351 | 0.006493146 | 0.019239583 |

**Supplementary Table 3.** Prediction of binding sites between EGR2 and SOX4

| TF | Score | Relative score | GENE | Start | End | Predicted sequence |
| --- | --- | --- | --- | --- | --- | --- |
| EGR2 | 13.163018 | 0.90651168 | SOX4 | 1722 | 1736 | CGCACACACACGCGC |
| EGR2 | 13.1425495 | 0.90612319 | SOX4 | 1704 | 1718 | TATACACACACGCAC |
