# Supplementary figures and images for: Temozolomide protects against the progression of glioblastoma via SOX4 downregulation by inhibiting the LINC00470‐mediated transcription factor EGR2
Source: CNS Neurosci Ther. 2023 Mar 29;29(8):2292–307. doi: 10.1111/cns.14181 (PMC10352878; doi:10.1111/cns.14181)

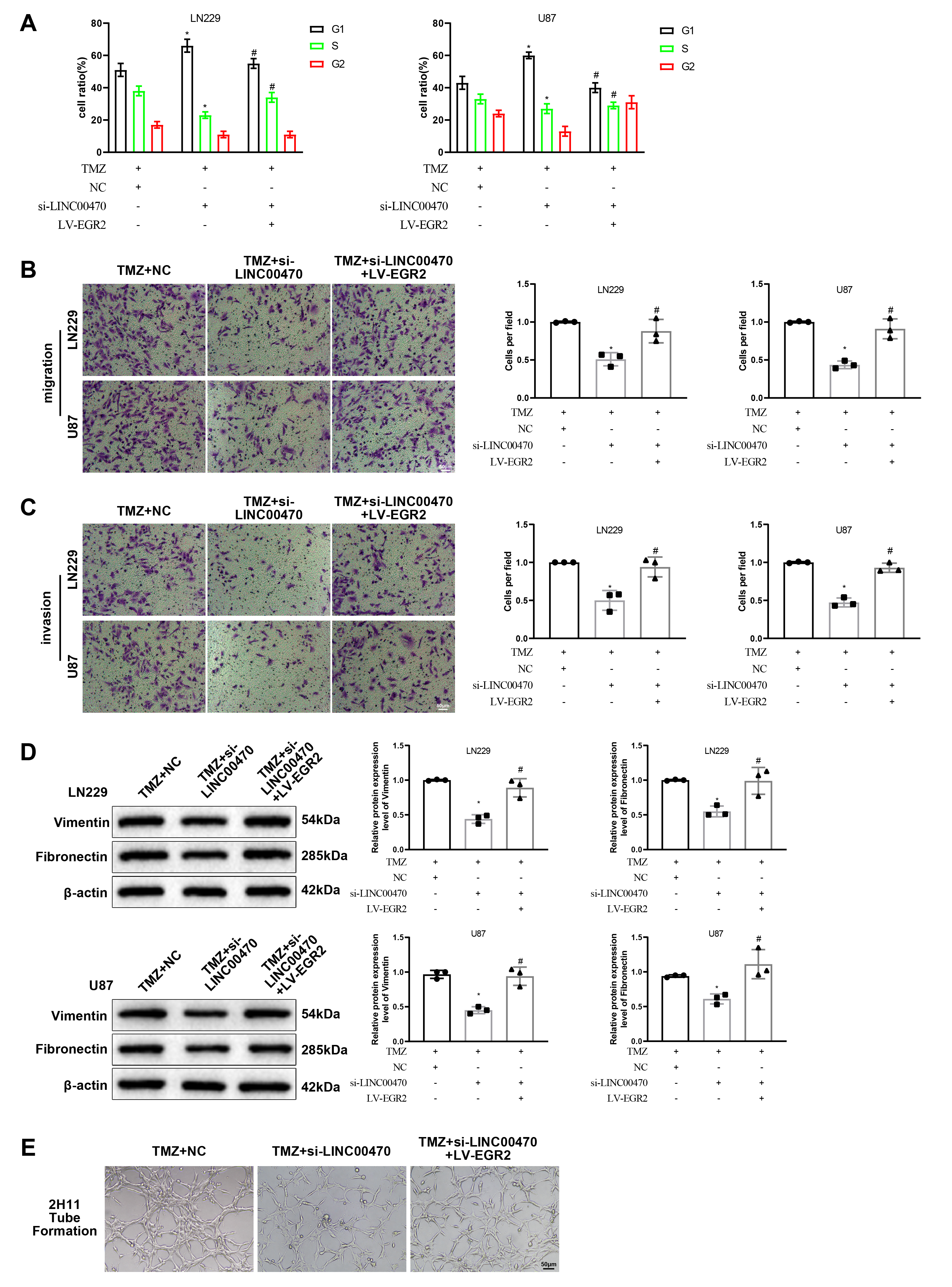

Supplement: Supplementary file 2 — Figure S1. [file CNS-29-2292-s002.jpg]

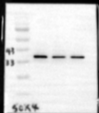

Supplement: Supplementary file 3 — Data S1. [file CNS-29-2292-s004.zip › CNS_14181_2F-SOX4.pdf]

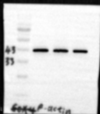

Supplement: Supplementary file 3 — Data S1. [file CNS-29-2292-s004.zip › CNS_14181_2F-β-actin.pdf]

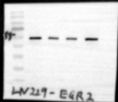

Supplement: Supplementary file 3 — Data S1. [file CNS-29-2292-s004.zip › CNS_14181_2I-LN229-EGR2.pdf]

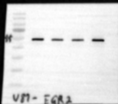

Supplement: Supplementary file 3 — Data S1. [file CNS-29-2292-s004.zip › CNS_14181_2I-U87-EGR2.pdf]

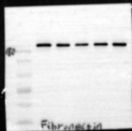

Supplement: Supplementary file 3 — Data S1. [file CNS-29-2292-s004.zip › CNS_14181_3d-LN229-Fibronectin.pdf]

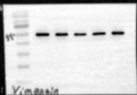

Supplement: Supplementary file 3 — Data S1. [file CNS-29-2292-s004.zip › CNS_14181_3d-LN229-Vimentin.pdf]

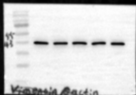

Supplement: Supplementary file 3 — Data S1. [file CNS-29-2292-s004.zip › CNS_14181_3d-LN229-β-actin.pdf]

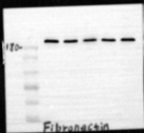

Supplement: Supplementary file 3 — Data S1. [file CNS-29-2292-s004.zip › CNS_14181_3D-U87-Fibronectin.pdf]

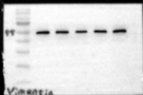

Supplement: Supplementary file 3 — Data S1. [file CNS-29-2292-s004.zip › CNS_14181_3D-U87-Vimentin.pdf]

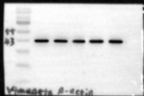

Supplement: Supplementary file 3 — Data S1. [file CNS-29-2292-s004.zip › CNS_14181_3D-U87-β-actin.pdf]

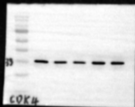

Supplement: Supplementary file 3 — Data S1. [file CNS-29-2292-s004.zip › CNS_14181_4F-CDK4.pdf]

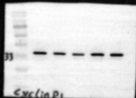

Supplement: Supplementary file 3 — Data S1. [file CNS-29-2292-s004.zip › CNS_14181_4F-CyclinD1.pdf]

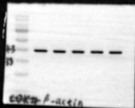

Supplement: Supplementary file 3 — Data S1. [file CNS-29-2292-s004.zip › CNS_14181_4F-β-actin.pdf]

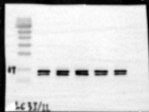

Supplement: Supplementary file 3 — Data S1. [file CNS-29-2292-s004.zip › CNS_14181_5C-LN229-LC3.pdf]

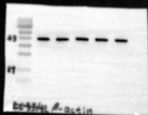

Supplement: Supplementary file 3 — Data S1. [file CNS-29-2292-s004.zip › CNS_14181_5C-LN229-β-actin.pdf]

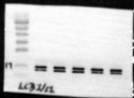

Supplement: Supplementary file 3 — Data S1. [file CNS-29-2292-s004.zip › CNS_14181_5C-U87-LC3.pdf]

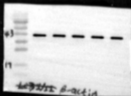

Supplement: Supplementary file 3 — Data S1. [file CNS-29-2292-s004.zip › CNS_14181_5C-U87-β-actin.pdf]

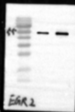

Supplement: Supplementary file 3 — Data S1. [file CNS-29-2292-s004.zip › CNS_14181_EGR2.pdf]

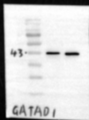

Supplement: Supplementary file 3 — Data S1. [file CNS-29-2292-s004.zip › CNS_14181_GATAD1.pdf]

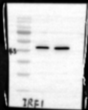

Supplement: Supplementary file 3 — Data S1. [file CNS-29-2292-s004.zip › CNS_14181_IRF1.pdf]

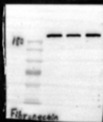

Supplement: Supplementary file 3 — Data S1. [file CNS-29-2292-s004.zip › CNS_14181_S-D-LN229-Fibronectin.pdf]

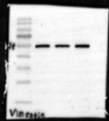

Supplement: Supplementary file 3 — Data S1. [file CNS-29-2292-s004.zip › CNS_14181_S-D-LN229-Vimentin.pdf]

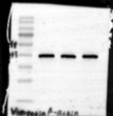

Supplement: Supplementary file 3 — Data S1. [file CNS-29-2292-s004.zip › CNS_14181_S-D-LN229-β-actin.pdf]

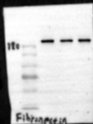

Supplement: Supplementary file 3 — Data S1. [file CNS-29-2292-s004.zip › CNS_14181_S-D-U87-Fibronectin.pdf]

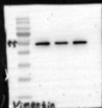

Supplement: Supplementary file 3 — Data S1. [file CNS-29-2292-s004.zip › CNS_14181_S-D-U87-Vimentin.pdf]

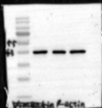

Supplement: Supplementary file 3 — Data S1. [file CNS-29-2292-s004.zip › CNS_14181_S-D-U87-β-actin.pdf]

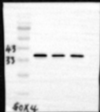

Supplement: Supplementary file 3 — Data S1. [file CNS-29-2292-s004.zip › CNS_14181_SOX4.pdf]

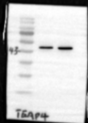

Supplement: Supplementary file 3 — Data S1. [file CNS-29-2292-s004.zip › CNS_14181_TEAD4.pdf]

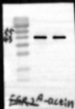

Supplement: Supplementary file 3 — Data S1. [file CNS-29-2292-s004.zip › CNS_14181_β-actin.pdf]
