# Supplementary material for: Temozolomide protects against the progression of glioblastoma via SOX4 downregulation by inhibiting the LINC00470‐mediated transcription factor EGR2
Source: CNS Neurosci Ther. 2023 Mar 29;29(8):2292–307. doi: 10.1111/cns.14181 (PMC10352878; doi:10.1111/cns.14181)
Supplement: Supplementary file 4 — Figure Caption [file CNS-29-2292-s001.docx]

**Figure S1.** EGR2 overexpression abrogated the protective effects of temozolomide and LINC00470 knockdown on glioblastoma
Notes: (A) Cycle distribution of LN229 and U87 cells was determined by flow cytometry. (B) Cell migration was measured by Transwell assay. (C) Cell invasion was evaluated by Transwell assay. (D) Vimentin and fibronectin protein expression in cells was examined by western blot. (E) Angiogenesis was assessed by matrix gel-based in vitro endothelial tube formation assay using 2H11 cells with different treatments. **p* < 0.05, compared with the TMZ + NC group; #*p* < 0.05, compared with the TMZ + si-LINC00470 group. Data were derived from three independent replicate experiments and compared using one-way analysis of variance with Tukey's test for post hoc multiple comparisons. EGR2, early growth response 2; TMZ, temozolomide; si, small interfering RNA; LV, lentiviral vectors for overexpression.
